# Risk Factors for Avian Influenza H9 Infection of Chickens in Live Bird Retail Stalls of Lahore District, Pakistan 2009–2010

**DOI:** 10.1038/s41598-018-23895-1

**Published:** 2018-04-04

**Authors:** Mamoona Chaudhry, Hamad B. Rashid, Angélique Angot, Michael Thrusfield, Barend M. deC Bronsvoort, Ilaria Capua, Giovanni Cattoli, Susan C. Welburn, Mark C. Eisler

**Affiliations:** 1 The University of Edinburgh. Deanery of Biomedical Sciences, Edinburgh Medical School, College of Medicine and Veterinary Medicine, The University of Edinburgh, 1 George Square, Edinburgh, Scotland EH8 9JZ UK; 2 Department of Clinical Medicine and Surgery, University of Veterinary and Animal Sciences, Abdul Qadir Jilani Road, Lahore, Pakistan; 3 OIE and National Reference Laboratory for Avian Influenza and Newcastle Disease, FAO Reference Centre for Animal Influenza and Newcastle Disease. OIE Collaborating Centre for Diseases at the Human Animal Interface. Istituto Zooprofilattico Sperimentale delle Venezie - Viale dell’Università 10, 35020 Legnaro (PD), Italy; 4 Royal (Dick) School of Veterinary Studies, University of Edinburgh, Easter Bush, Roslin, Midlothian, EH25 9RG Scotland UK; 5 Roslin Institute at the R(D)SVS, University of Edinburgh, Easter Bush, Roslin, Midlothian, EH25 9RG Scotland UK; 6 One Health Center of Excellence for Research and Training, University of Florida, Gainesville, FL 32611 USA; 7 Animal Production and Health Laboratory, Joint FAO/IAEA Division for Nuclear Applications in Food and Agriculture, International Atomic Energy Agency, Seibersdorf, Austria; 8 Zhejiang University, – University of Edinburgh Joint Institute, Zhejiang University, International Campus,718 East Haizhou Road, Haining, Zhejiang, 314400 P.R. China; 9 Bristol Veterinary School, University of Bristol, Langford House,Langford, Bristol, BS40 5DU UK; 10 Department of Epidemiology and Public Health, University of Veterinary and Animal Sciences, Abdul Qadir Jilani Road, Lahore, Pakistan

## Abstract

This study was conducted to identify risk factors associated with AIV infections in live bird retail stalls (LBRS) in Lahore District, Pakistan. A cross-sectional survey of LBRS was conducted from December 2009-February 2010 using two-stage cluster sampling based on probability proportional to size. A total of 280 oropharyngeal swab sample pools were collected from 1400 birds in 8 clusters and tested by qRT-PCR for the matrix (M) gene of type A influenza virus and HA gene subtypes H9, H5 and H7. Thirty-four (34) samples were positive for the M gene, of which 28 were also positive for H9. No sample was found positive for H5 or H7. Data for 36 potential risk factors, collected by questionnaire, were analyzed by survey-weighted logistic regression and prevalence odds ratios (OR) for associated risk factors were calculated. A final multivariable model identified three risk factors for H9 infection in LRBS, namely obtaining birds from mixed sources (OR 2.28, CI_95%_: 1.4–3.7), keeping birds outside cages (OR 3.10, CI_95%_: 1.4–7.0) and keeping chicken breeds other than broilers (OR 6.27, CI_95%_: 1.7–23.2). Sourcing birds from dealers/wholesalers, keeping birds inside cages and avoiding mixing different breeds in cages could reduce the risk of H9 infections in LRBS.

## Introduction

Live bird markets (LBM) are an important component of traditional poultry marketing systems in many developing countries. The characteristics of LBM have not been particularly well studied, but are known to vary from region to region depending upon the needs of the local customers^1,2^.

These LBM, also known as ‘wet markets’ have been recognized as important for the maintenance and viral re-assortment of avian influenza viruses (AIV)^3–5^. The risks associated with these markets are exacerbated, where large numbers of poultry are maintained with poor biosecurity, as in many Asian countries^6^. Wet markets are thought to be a missing link in the epidemiology of AIV and due to the low-level biosecurity measures, these markets can provide a promising environment for perpetuation of AIV among various species, including humans^7,8^. The H5N1 outbreak in Hong Kong in 1997 left no doubt about the role of these markets as a source of novel infection for humans^9–12^.

In Pakistan, live bird retail stalls (LBRS) are the main outlets for fresh poultry meat, but their characteristics have not been well studied. Broiler business mainly operates through commission agents, feed dealers, and butchers^13^. Poultry farmers sell their products through main wholesale markets, small town markets and directly from farm^14^. Individual stalls are scattered throughout the urban and rural areas; however, in some areas several stalls are located in the same vicinity and collectively comprise a large LBM. These stalls mostly sell commercial broiler and indigenous chicken breeds, but in some markets, quails, turkeys, house sparrows and ducks are also sold alongside broiler birds^15^. The risk of AIV is higher in these multi-species stalls due to poor biosecurity. Mixing of birds from different sources may also play a role in spread of AIV in these stalls^16^.

Because these LBRS could serve as reservoir of AIV, a major threat for animal and public health, identification of local risk factors is important. The most important known factors are: poultry trading pattern^2,17^, mixing newly arrived birds in cages already having leftover birds from previous batches^4,18^, mixing species in the same cages, source of purchase of chickens^4^, sharing the equipment used for slaughtering, feeding, watering, and weighing the birds^19^, the number of birds sold per day^4,18,20^, movement of poultry through LBM^2,21^. To reduce the spill-over of these viruses from LBRS to susceptible avian and human population, these risk factors should be controlled. To our knowledge, no work has been done to quantify risk factors associated with infection of AIV in LBRS of Lahore District, Pakistan using classical epidemiological techniques.

We previously demonstrated the presence of avian influenza A subtype H9 at an overall prevalence of 10% in gallinaceous poultry in LBM of Lahore District, Pakistan, with individual town prevalences varying from 2.85% to 17.1%^15^. We also very briefly summarized a number of characteristics of individual LBRS of potential relevance for AIV infection, but made only a cursory attempt to relate these to infection status. Here we report these characteristics in detail and relate them to AIV H9 infection status in individual LBRS in univariable and multivariable risk factor analyses. Improved knowledge of the main risk factors for AIV in LBM infection should help understand the epidemiology and might improve control of AIV in live bird market systems.

## Results

A cross-sectional study was conducted in LBRS of Lahore District. Eight clusters were selected from a list of 9 towns of Lahore using a two-stage probability proportional to size (PPS), cluster sampling method. From each cluster, 35 LBRS were included as elementary units. A total of 280 LBRS were visited and a pool of 5 swab samples was collected from each LBRS. Thirty-four (34) of these 280 composite swab samples were positive by qRT-PCR for type A influenza (M gene). Out of 34 M gene positive samples, 28 were positive for subtype H9. None of these 34 samples was positive for H5 or H7 subtypes. Six samples positive for M gene but negative for H9, H5 and H7 were not further subtyped due to limited resources.

### Prevalence of AIV

**Figure 1:**
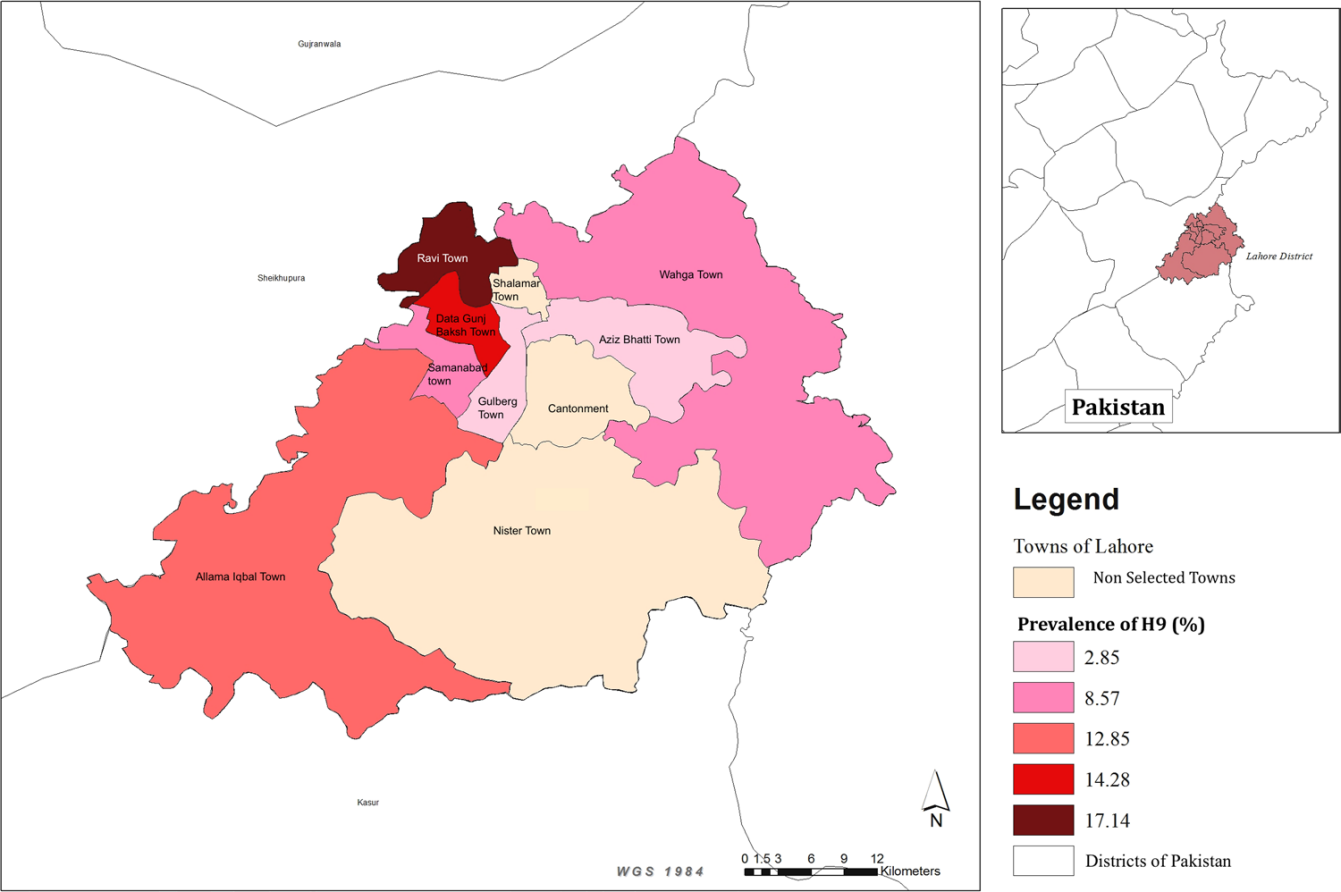
Prevalence estimates of H9 infection in selected towns of Lahore. Map was created using ArcGIS version 10 (http://www.esri.com) by the first author (MC).

As previously reported^15^, the overall virus prevalence of H9 virus was 10.0% (CI_95%_: 5.61%–14.39%). The highest individual town-level prevalence was recorded in Ravi town (17.1%; CI_95%_: 4.4%–29.8%), while the lowest prevalence was seen in Gulberg and Aziz Bhatti towns (2.8%; CI_95%_: 2.7–8.4%) (Fig. 1). The design effect (DE) was calculated to be 1.56 and intra-cluster correlation coefficient (rho) was 0.016 for PPS sampling.

Univariable analysis (Table 1) identified six potential risk factors based on the selection criterion (i.e. p < 0.25) that was used to develop the multivariable model.

**Table 1:** Univariable analyses of potential risk factors for the presence of AIV H9 in the LBRS of Lahore District, Pakistan

Variable	Response level	Range	Positive for H9`	Negative for H9	Odds Ratio (OR)	CI_95%_ for OR	P-value*
Number of cages in the stall	Number	1−8	28	152	1.33	0.85−2.08	0.252
Total number of birds kept in stall	Number	14−1000	28	152	1.00	1.00−1.01	0.338
Number of birds sold per day	Number	10−1000	28	152	1.00	0.99−1.00	0.411
Chicken breeds other than broiler in the stall (Indigenous, Fayoumi, or mixed breeds)	Yes	—	20	73	7.97	2.21−28.65	0.019
	No	—	8	179	Reference		
Source of birds for stall	Obtained from mixed source	—	18	76	4.40	3.15−6.16	< 0.001
	Dealer/wholesaler	—	10	176	Reference		
Market vehicle picks up birds from farm	Yes	—	1	12	0.44	0.04−4.32	0.510
	No	—	27	240	Reference		
Vehicle disinfected between deliveries	Yes	—	1	2	0.57	0.03−10.73	0.721
	No	—	27	250	Reference		
Adding new birds to cages already holding birds	Yes	—	25	194	3.73	0.70−19.86	0.173
	No	—	3	58	Reference		
Stall completely cleaned and disinfected within last 30 days	Yes	—	8	72	0.84	0.31−2.29	0.759
	No	—	20	180	Reference		
Keeping birds in the stalls during cleaning	Remain in stall	—	3	7	2.92	0.64−13.28	0.214
	Moved to elsewhere	—	25	245	Reference		
Wild birds around stall	Usually	—	27	243	1.00	0.60−1.66	0.998
	Rarely	—	1	9	Reference		
Rodents seen in the stall	Usually	—	11	45	2.67	1.46–4.86	0.018
	Rarely	—	17	207	Reference		
Avian mascot/pet bird in stall	Yes	—	3	29	1.00	0.35–2.36	0.872
	No	—	25	223	Reference		
Birds kept at home	Yes	—	7	57	1.06	0.40–2.80	0.896
	No	—	21	195	Reference		
Sick birds in stall	Moved to separate cage	—	1	2	2.80	0.03–223.1	0.663
	No special handling	—	26	246	0.41	0.03–4.25	0.489
	Moved to separate area	—	1	4	Reference		
Disposal of dead birds	Sold	—	26	228	0.49	0.11–2.23	0.399
	Trash pick up	—	2	24	Reference		
Housing of birds	Keeping some birds outside cages	—	7	23	3.36	1.39–8.13	0.036
	All birds housed inside cages	—	21	229	Reference		
Stray dogs accessing stall	Yes	—	19	164	1.12	0.57–2.17	0.746
	No	—	9	88	Reference		
Washed gizzards	Dip in bucket of water	—	26	220	2.80	0.29–26.58	0.403
	Separately under tap	—	2	32	Reference		
Another nearby stall	Yes	—	25	214	2.30	0.30–17.3	0.449
	No	—	3	38	Reference		
Number of people visiting stall	Number	12–700	152	28	1.00	0.99–1.01	0.279
Stall owned by respondent	Yes	—	27	242	1.04	0.45–2.38	0.924
	No	—	1	10	Reference		

^*^P-value based on Wald statistics.

### Factors associated with the prevalence of H9 in LBRS

In the univariable analysis, four variables showed strong association (i.e. p < 0.05) with AIV H9 prevalence. Among them, obtaining birds from mixed sources for stall had the highest level of statistical significance (p < 0.001). Rodents seen in the stall (p = 0.018), having chicken breeds other than broiler in the cages (p = 0.019), and keeping some birds outside cages (p = 0.036) were also positively associated with H9 infection. Two variables, namely keeping birds in stalls during cleaning (p = 0.214) and adding new birds to cages already holding birds (p = 0.173), showed weak positive associations (p < 0.25) with H9 infection in these stalls. Sixteen variables (including number of cages in the stall, total number of birds kept in stall, number of birds sold per day, market vehicle picks up birds from farm, stall completely cleaned and disinfected within last 30 days, vehicle disinfection between deliveries, wild birds around stall, avian mascot/pet bird in stall, birds kept at home, sick birds in stall, disposal of dead birds, stray dogs accessing the stall, washed gizzards, another nearby stall, number of people visiting stall per day, and stall owned by respondent) showed no association (p > 0.25) with prevalence of H9 in the LBRS (Table 1). Analysis for 14 variables (Days of week stall remained open, presence of spent layers, presence of broiler roaster, presence of ducks, presence of guinea fowl, presence of turkey, presence of pheasants, presence of quails, presence of chukars/partridges, presence of geese, presence of pea fowl, presence of pet birds, stray cats in the stall, and washing instrument before slaughter) could not be conducted due to zero cell values in 2 × 2 contingency tables.

The final multivariable model identified one variable, the source of birds for the stall, i.e. obtaining birds from mixed sources rather than from a dealer/wholesaler (OR 2.28, CI_95%_: 1.4–3.7; p = 0.029), as an independent risk factor strongly associated with H9 prevalence in these LBRS. Two variables, namely having chicken breeds other than broiler (e.g. indigenous/Fayoumi or mixed breeds) in the stall (OR 6.27, CI_95%_: 1.7–23.2; p = 0.051), and not housing all birds, i.e. keeping some birds outside cages, (OR 3.10, CI_95%_: 1.4–7.0; p = 0.052) were moderately associated with H9 prevalence (Table 2).

**Table 2:** Risk factors associated with AIV H9 infection in LBRS of Lahore District, Pakistan, based on a forward stepwise selection process of selected variables with p ≤ 0.25 in univariable analysis.

Potential Risk Factors	Odds ratio (OR)	Confidence Interval (CI_95%_) for OR	P-value
Source of birds	2.28	1.4–3.7	0.029
Housing of birds	3.10	1.4–7.0	0.052
Chicken breeds other than broiler	6.27	1.7–23.2	0.051

## Discussion

Live bird markets are an important source of AIV because they bring together numerous bird species (e.g. broiler chickens, ducks, geese, turkeys etc.) in a high-density setting, an ideal environment for virus genome re-assortment and interspecies transfer^22^. These markets have been termed potential “hotspots” of influenza A viruses, but their role in human infection has not been firmly established^21^.

As previously reported, the overall prevalence of H9 in LBRS in Lahore was 10%^15^, higher than the 2.5% prevalence reported from China^23^, but lower than the 16.5% prevalence reported in Bangladesh^5^. Differences in these prevalence estimates in various countries could reflect diverse risk factors e.g. practices of handling, keeping and slaughtering of birds in LBM or the frequency of virus circulation, or may be due to differences in survey methodologies. In Korea, H9N2 viruses were the most abundant AIV isolated from chicken, ducks, and doves from LBMs, accounting for 56% of isolates^24^. Similarly, in the current study, 82.4% (28/34) of AIV positive pools belonged to the H9 subtype. Our results confirmed the circulation of H9 in LBRS of Lahore District, and emphasize the need for regular planned surveillance of these stalls to monitor activity of AIV.

No positive samples for H7 or H5 were detected. This might be due to the on-going vaccination strategy at broiler, layer and breeder farms, or may be by chance. These results indicate near absence of these two subtypes from commercial poultry farms supplying birds to Lahore District.

The design effect (DE) for the PPS cluster sampling method used was 1.56, indicating that the variance of the prevalence estimated by the current study would probably be similar to that calculated by simple random (SR) sampling. The intra-cluster correlation coefficient (rho) of 0.016 indicated that there is much higher heterogeneity within the cluster compared with between clusters^25^. These values of DE and rho confirmed that there was very low effect of the clustering in the data and that only a small difference in the point prevalence estimate would be expected when comparing the PPS clustering sampling method used here with simple random sampling method.

Live bird retail stalls obtaining birds from mixed sources (e.g. other live poultry markets, auction markets, farm/individual producers) were more likely (OR = 2.28, CI_95%_: 1.4–3.7; p = 0.029) to become infected with H9 than LBRS that purchased birds only from a single source (dealer/wholesaler). Birds coming from mixed sources have more chances of bringing infection to a stall than birds coming from a single source. The source of birds for stall (small-scale backyard farmers, commercial farms, or combination, auction markets etc.) was not proven to be a risk factor for presence of AIV in Indonesia^4^. However, that study used environmental samples rather than direct swabbing of poultry. Moreover, purchase of live birds/eggs directly from farm also poses a threat to commercial poultry^26^. The movement of individuals and vehicles between farms and markets might spread the virus mechanically. Our results suggest that purchasing birds from the dealer/wholesaler rather than mixed sources would reduce the risk of H9 AIV infection in retail stalls.

The results of the current study showed that H9 infection was more likely in LBRS with some birds outside cages (OR = 3.10, CI_95%_: 1.4–7.0; p = 0.052) than LBRS that housed all birds inside cages. Hence, we suggest that always housing birds inside cages is safer in terms of H9 infection. Birds outside the cages have more chance to come in contact with terrestrial wild birds (e.g. house sparrow)^27,28^ and thus to become infected with AIV.

Having chicken breeds other than broiler in the stall e.g. indigenous/Fayoumi or mixed breeds in the stall was positively associated with the likelihood of H9 infection (OR = 6.27, CI_95%_: 1.7–23.2; p = 0.051). Presence of these other poultry breeds in LBM has been reported as a risk factor for environmental contamination with AIV^29^. Indigenous chicken breeds are usually reared in village backyards with low biosecurity and therefore more likely to come in contact with infected wild birds^30^. These birds can introduce AIV infection through cross-contamination when sold to urban LBRS for cash and mixed in the same cage with commercial broilers birds, which are reared on commercial farms under high biosecurity^16^. Hence, mixing of different breeds may increase the likelihood of dissemination and genetic reassortment of influenza viruses^3^ due to variable pathogenicity of AIV in indigenous and commercial breeds^31^. Differences in the lethality of HPAIV infection among various lines or breeds of chicken have been described previously^32^.

Awareness of LBRS owners about susceptibility of different breeds to AIV could be useful in minimizing the spread.

Adding new birds to cages already holding birds was weakly associated with H9 infection in the univariable analysis. In multivariable analysis, this variable was dropped, being non-significant compared with other predictor variables in the model. Nevertheless, it is biologically plausible and the risk of H9 infection could be increased if new birds were added to cages already housing birds.

Birds might become infected and shed AIV within 24 hours in an infected market. If held overnight, these infected ‘leftover’ birds might spread virus to new birds by the following day^18^. Introducing a rest day during which LBM stalls are completely depopulated of these ‘leftover’ birds and disinfected, has been reported as an effective interventional strategy in controlling further spread^33^.

Presence of rodents in stall showed a significant positive association in the univariable analysis, but was dropped in the multivariable model due to non-significance. Nevertheless, implementation of a pest control program in LBRS might be helpful to control AIV by this potential route of transmission.

No association was found between various other factors (keeping birds in the stalls during cleaning, number of cages in the stall, total number of birds kept in stall, number of birds sold per day, market vehicle picks up birds from farm, stall completely cleaned and disinfected in last 30 days, vehicle disinfected between deliveries, wild birds around stall, avian mascot/pet bird in stall, birds kept at home, sick birds in stall, disposal of dead birds, stray dogs accessing the stall, washed gizzards, another nearby stall, number of people visiting stall per day, and stall owned by respondent) and H9N2 infection in the current study. However, these factors were reported as significant determinants in other studies^2,4,9,18–21,33–39^.

Our cross-sectional study was only a snapshot of the situation over a specified period of time and, hence causality between risk factors and outcome could not be deduced. Detailed longitudinal studies would be required to establish causality. In addition, as a comprehensive database of LBRS in different towns of Lahore was not available, the sample size for the current study was constructed from a list of administrative towns in Lahore and approximate total numbers of LBRS in each town. Two-stage cluster sampling with probability proportional to size with replacement was used to overcome this lack of a complete sampling frame and to ensure economic feasibility. We acknowledge that bias could occur because available population data were not up-to-date and potentially inaccurate.

Lastly, some questions may have been interpreted subjectively by the respondents. For instance, some respondents who did not personally observe the wild birds and rodents in their stalls, might have answered “No” in spite of other indications of their presence. Moving sick birds to separate cages, may also have been biased towards the null, because stall owners may have been influenced by Hawthorne effect^40,41^ i.e. their answers are biased by their presumption of the researcher’s expectations. In some instances, recall bias might also have influenced the respondents’ answers.

## Conclusion

This study confirmed the presence of H9 infection in LBRS in Lahore and supported the hypothesis that H9 virus is circulating and possibly persisting in these LBRS. A major aim of this study was to facilitate control of AIV in different compartments of the poultry production system in Pakistan by providing baseline data for large-scale surveillance of LBRS and the results could be helpful in developing a risk-based approach for AIV risk reduction in LBRS. Moreover, implementation of recommendations for sourcing birds only from dealers/wholesale markets, keeping birds inside the cages only and avoiding mixing of different chicken breeds in the same cage could reduce the immediate risk of H9 infections in these stalls.

## Methods

A cross-sectional survey of LBRS in Lahore District was conducted during December 2009-February 2010. The sampling plan and survey methodology have been previously reported briefly^15^. Two-stage cluster sampling with probability proportional to size with replacement (PPSWR) was used^24,42^. Lahore, the capital city of Punjab Province, has nine administrative towns and one cantonment area. These towns were considered as areal units or clusters^43^ due to their distinct geographical boundaries. The sampling frame was constructed from the list of nine administrative towns of Lahore with approximate total numbers of LBRS in each town (overall total 14,950) provided by PLDDD (see supplementary material). The sample size was calculated using C-Survey, version 2.0^44^. Towns of Lahore District were selected as primary sampling units or clusters (PSU), within which individual LBRS were elementary units. The sample size calculation required selection of eight PSU; seven towns were selected, of which one (Allama Iqbal Town) was selected twice owing to replacement. Within the selected PSU, a fixed number (35) of LBRS were selected as elementary units systematically i.e. a starting point was chosen arbitrarily within the commercial area containing the retail shops. Hence the study population comprised 280 LBRS located among the seven towns of Lahore District selected as PSU. All birds in a LBRS were treated as a single flock because they were kept under the same conditions. At each LBRS, individual swabs from the oropharyngeal tracts of five apparently healthy live birds (broiler and indigenous chicken), selected at the arbitrary choice of stall owner, were obtained and pooled. Each pool was considered as a single composite sample.

A questionnaire including both closed and semi-closed questions about potential risk factors for H9 infection was pretested on 10 non-selected LBRS in Lahore District and completed during face-to-face interviews with owners /managers of the selected LBRS. Locations of LBRS were recorded with a hand-held global positioning system (Garmin, Olathe, KS, USA).

Pooled oropharyngeal swab samples (n = 280) collected from 1400 birds and were tested by qRT-PCR for matrix (M) gene of type A influenza viruses^45^ first, followed by subtyping for HA gene^46^. Characterization by qRT-PCR for influenza type A and for subtype H5, H7 and H9 was carried out under bio-containment level 2 (BSL-2) at the OIE and National Reference Laboratory for Avian Influenza and Newcastle Disease, FAO Reference Centre for Animal Influenza and Newcastle Disease, Istituto Zooprofilattico Sperimentale delle Venezie, Padua, Italy. All positive H9 samples were further analyzed by virus isolation test and were characterized by sequencing for 6 gene segments (hemagglutinin, neuraminidase, nonstructural, matrix, and polymerase basic 1 and 2). The results of the sequence analysis of isolated viruses have been published previously^47^.

Animal sampling was conducted in strict accordance with the national and international animal care guidelines [Convention of European Council no. 123 and National guidelines (DLgs 116/92)] and approved by Istituto Zooprofilattico Sperimentale delle Venezie’s Ethics Committee. There was no requirement for ethical review to sample the birds as undertaken in this study in Pakistan at the time of sampling. Registered and trained veterinarians (MC & HBR) collected samples following standard protocol. Permission was sought from the local Veterinary Officers. Informed consent was obtained from each stall owner to collect swab samples and related information. There were no non-responders because all the owners gave permission to collect oropharyngeal swabs and answered all of the questions in the questionnaire.

### Data Analysis

All statistical analyses were conducted using R software^48^. Collected data were stored in digital form using EpiData software 3.1^49^. Stored data were exported in dBase and Excel format for further processing and analysis in ArcGIS 10 (Geographical Information System, ESRI System, Redlands, CA, USA) and statistical analysis using the R statistical software. Validation of data was done by crosschecking each original hard copy form with digital records.

Point estimates of virus prevalence in LBRS with associated confidence intervals (CI_95%_) were calculated using *svy* command of survey package in R software^50^. Thirty-six potential risk factors were examined for association with outcome (LBRS either positive or negative for H9) by measuring the odds ratio (OR). The survey weighted logistic regression was used to fit the univariable and multivariable models, and prevalence OR with CI_95%_ for each explanatory variable were calculated using the survey package in R software^50^. Standard model building methodologies were adopted to select each explanatory variable^51,52^. All tests were two-sided. All variables associated with AIV infection in LBRS with p < 0.25 in the univariable analysis were included in the multivariable logistic regression model. After univariable analysis, selected variables were checked for collinearity using spearman rank correlation. Phi correlation coefficient (*ϕ)* for binary categorical variables was calculated^53^. If there was strong positive correlation (*ϕ* > 0.5) between the variables, the more clinically important and biologically plausible variable from pairs of correlated variables was chosen for the multivariable model.

A forward stepwise variable–selection strategy was used to construct a final model at a specified α level (p < 0.05). Variables were retained or removed from the model after considering the Wald statistics with a p-value of 0.05 and comparison of each estimated coefficient with the coefficient from the model containing only the variable. If a variable was excluded from the model, the estimated coefficients for the remaining variables were also compared to those from the full model^51,52^. After inclusion and/or exclusion of each variable, the new model was compared with the previous one by the Akaike Information Criterion (AIC) for a fitted parametric model^52^.

Paper maps at a scale of 1:75,000 were obtained from Survey of Pakistan for Lahore District published in 2002 and map showing town boundaries of Lahore District was downloaded from the website of City District (http://www.lahore.gov.pk/city-government/lahore-map.aspx). These maps were digitally scanned and georeferenced using ArcGIS 10 (ESRI, Redlands, CA, USA). Map of spatial distribution of LBRS in different towns of Lahore District was created by the first author.

### Data availability

The datasets generated during and/or analyzed during the current study are available from the corresponding author on reasonable request.

## Electronic supplementary material

Supplementary Material
