# Supplementary material for: Risk Factors for Avian Influenza H9 Infection of Chickens in Live Bird Retail Stalls of Lahore District, Pakistan 2009–2010
Source: Sci Rep. 2018 Apr 4;8:5634. doi: 10.1038/s41598-018-23895-1 (PMC5884806; doi:10.1038/s41598-018-23895-1)
Supplement: Supplementary file 1 — Supplementary Material [file 41598_2018_23895_MOESM1_ESM.doc]

# Risk Factors for Avian Influenza H9 Infection of Chickens in Live Bird Retail Stalls of Lahore District, Pakistan 2009-2010

**Authors:** Mamoona Chaudhry^1*^, Hamad B. Rashid^2^, Angélique Angot^3^, Michael Thrusfield^4^, Barend M. deC. Bronsvoort^5^, Ilaria Capua^3,6^, Giovanni Cattoli^3,7^, Susan C. Welburn^1,8^, Mark C. Eisler^9^

**Supplementary Material**

1. Number of LBRS in each town used for sampling

*Summary Table: Sampling frame for selection of clusters (towns of Lahore)*

| **Name of Town**  **1^st^ Stage** | **Total population of LBRS** | **No. of LBMS Sampled**  **2^nd^ Stage** |
| --- | --- | --- |
| Ravi | 1550 | 35 |
| Data Gunj Bakhsh | 1300 | 35 |
| Samanabad | 2200 | 35 |
| Gulberg | 1000 | 35 |
| Allama Iqbal | 1300 | 70 |
| Aziz Bhatti | 1900 | 35 |
| Wagha | 2000 | 35 |
| Shalimar | 1800 | (Not selected) |
| Nister | 1900 | (Not selected) |
| **Total *(N)*** | **14950** | **280** |

# 2. Qustionnaire For Study Of Risk Factors Of AIV Prevalence On Live Bird Retail Stalls Of Lahore District

**This section must be filled in before completing the questionnaire**:

| Date: | Questionnaire No: |
| --- | --- |
| Town Name: | |
| Area Name: | Market Name: |
| Latitude: | Longitude: |
| Name of Respondent: | Name of Stall: |

1. How many days of a week, this stall remains open?......**Seven □ less than 7 □**
2. How many cages do you have in your stall? ……..........… _______________
3. Approximately how many total birds, you keep in your stall? ..…. ____ birds
4. On average, approximately how many birds are sold per day? …._____ birds

Which type of birds you sell on your shop?

1. Spent laying hens (table egg layers) ............................................. **Yes** □ **No** □
2. Broilers or roasters ........................................................................ **Yes** □ **No** □
3. Other chickens (e.g., Indigenous, Fayoumi, or mix)....…......….....**Yes** □ **No** □
4. Ducks ............................................................................................ **Yes** □ **No** □
5. Guinea fowl ................................................................................... **Yes** □ **No** □
6. Turkeys ......................................................................................... **Yes** □ **No** □
7. Pheasants ....................................................................................... **Yes** □ **No** □
8. Quail .............................................................................................. **Yes** □ **No** □
9. Chukors/partridges ........................................................................ **Yes** □ **No** □
10. Geese ............................................................................................. **Yes** □ **No** □
11. Peafowl ......................................................................................... **Yes** □ **No** □
12. Pet birds (parrots, parakeets, canaries, etc) ................................... **Yes** □ **No** □
13. How do you get birds for your stall?..................................................................

…………………………………………**Dealers or wholesalers** □ **Mix source** □ (other live bird market, wholesale market, auction or individual farm producer)

1. Does market vehicle pick up birds from the farms......................... **Yes** □ **No** □
2. Are vehicles disinfected between deliveries? ................................. **Yes** □ **No** □
3. Do you add newly arriving birds to cages that already contain birds?*……...*………………………………………...…………..... **Yes** □ **No** □
4. Have you completely cleaned and disinfected this stall including floors, walls, cages, AND ceilings at one time in the last 30 days? …………………………………….………................................ **Yes** □ **No** □
5. What do you do with the birds when you clean and disinfect the stall?………... ……………………………………... **Birds/animals remain in the market as usual □ Birds/animals moved elsewhere in the market □**
6. How often do you see wild birds around your stall? … **Usually** □ **Rarely** □
7. How often do you see wild rodents (rats and mice) in your stall? ........................................................................................ **Usually** □ **Rarely** □
8. Does this market have a resident flock or avian mascot/pet? ..... **Yes** □ **No** □
9. Do you keep birds at home? ....................................................... **Yes □ No □**
10. What do you usually do when sick birds are identified in this stall?.....................**Move to a separate area from the healthy birds □ Move to a separate cage in the same area as healthy birds □ No special handling □**
11. How does this stall usually dispose of dead birds and offal? *(Tick one only)* ….……...……………….….……….. .......................**Sold □ Trash pick up □**
12. How are the birds are housed in stall *………………………………………* ……...........… **keeping some birds outside cages □ All birds inside cages □**
13. Do stray dogs have access to stall? ............................................... **Yes □ No □**
14. Do stray cats have access to stall? ................................................ **Yes □ No □**
15. How you wash gizzard of slaughtered birds?…………………….. …………………...….…...**dip in bucket of water □ separately under tap □**
16. Do you wash instrument for slaughtering one birds after slaughtering other bird? ………………………………………….……......……….. **Yes** □ **No** □
17. Do you have any other stall nearby? ……..………….………….. **Yes** □ **No** □
18. How many people visit your stall in a day? ….….............. _____________
19. Do you own the stall?.................................................................**......Yes** □ **No** □
